# Indecomposability of entanglement witnesses constructed from symmetric measurements

**DOI:** 10.1038/s41598-022-14920-5

**Published:** 2022-06-24

**Authors:** Katarzyna Siudzińska

**Affiliations:** Institute of Physics, Faculty of Physics, Astronomy and Informatics, Nicolaus Copernicus University in Toruń, ul. Grudziądzka 5, 87–100 Toruń, Poland

**Keywords:** Information theory and computation, Quantum physics

## Abstract

We propose a family of positive maps constructed from a recently introduced class of symmetric measurements. These maps are used to define entanglement witnesses, which include other popular approaches with mutually unbiased bases and mutually unbiased measurements. A particular interest is given to indecomposable witnesses that can be used to detect entanglement of quantum states with positive partial transposition. We present several examples for different number of measurements.

## Introduction

Quantum entanglement is an essential resource that plays a fundamental role in quantum information processing, quantum communication, quantum computing, and other modern quantum technologies^21,31^. In particular, any bipartite entangled state enhances the teleportation power^29^ and displays hidden nonlocality^30^. The usefulness of quantum tasks usually increases with the amount of entanglement^2,41,42^. Characterization of entangled states is essential both in theory and practice. However, the problem of distinguishing between separable and entanged states remains open; in fact, it is NP-hard^14^.

For qubit-qubit and qubit-qutrit systems, the necessary and sufficient separability condition is given by the celebrated Peres-Horodecki positive partial transposition (PPT) criterion^19,32^. In higher dimensions, this condition is only necessary, which first was shown for a qutrit-qutrit system^19^. More refined detection methods include the computable cross-norm or realignment (CCNR) criterion^4,6,18,34^, the correlation matrix criterion^9,10^, the local uncertainty relationship criterion^16^, the reduced density matrix criterion^3^, and the covariance matrix criterion^13^.

Another approach to entanglement detection is through entanglement witnesses, which are Hermitian block-positive (but not positive) operators. Hence, any such operator is positive on separable states, and a state $\rho$ is separable if and only if $\mathrm {Tr}(\rho W)\ge 0$ for every entanglement witness *W*. All entangled states have witnesses that detect them^43,44^. In other words, if $\rho$ is entangled, there exist a (non-unique) witness *W* of its entanglement such that $\mathrm {Tr}(\rho W)<0$. The problem lies in finding a suitable witness for a given state. The advantage of choosing entanglement witnesses over other detection methods is that non-separability of a state is decided upon calculating expectation values of *W* in that state. Therefore, it requires less information than a full state tomography, which also means less experimental devices and fewer measurements performed.

There exists a special class of witnesses that can detect quantum states with positive partial transposition, also known as bound entangled states^17,20,24,25,44^. They are called indecomposable because they cannot be decomposed into $W=A+B^\Gamma$ with positive *A* and *B*, where $\Gamma$ is a partial transposition. There is no general construction method for such operators, and it is often hard to determine whether a witness is decomposable or not. However, several classes of indecomposable entanglement witnesses have been found, like the ones related to the well-known realignment or computable cross-norm (CCNR) separability criterion^5,6,35^ and covariance matrix criterion^12,13,26^, as well as their generalizations^37,38^.

In the construction of entanglement witnesses, one often uses mutually unbiased bases (MUBs). Orthonormal bases in ${\mathbb {C}}^d$ are mutually unbiased if and only if the transition probability between any two vectors that belong to different bases is constant^11^. In ref.^8^, the authors used MUBs to define a new class of witnesses and analyzed their properties in $d=3$. This construction has been generalized in many ways. Li et al. introduced analogical operators for mutually unbiased measurements (MUMs)^27^ and symmetric informationally complete measurements (SIC-POVMs)^28^. Wang and Zheng^45^ considered the MUB-based witnesses in composite systems with different dimensions. Hiesmayr et al.^15^ showed that inequivalent and unextendible sets of MUBs are sometimes more useful to detect entanglement, whereas Bae et al.^1^ found that more than $d/2+1$ MUBs are needed to identify bound entangled states. The MUMs encompassing a full range of purity were shown to detect entanglement with as little as two measurement operators^36^. Recently, it has also been proven that the witnesses constructed from MUMs belong to the class based on the CCNR criterion^40^.

In this paper, we construct a class of entanglement witnesses based on symmetric measurements introduced in ref.^39^. These measurements include mutually unbiased bases, mutually unbiased measurements, and symmetric informationally complete POVMs as special cases. Therefore, they can be used to define broader families of positive maps and witnesses of quantum entanglement. In any finite dimension $d>2$, one has at least four informationally complete sets of symmetric measurements. Their ability to detect quantum entanglement has already been shown^39^. We recall the definition and basic properties of such operators, together with the construction method. Next, we use a (not necessarily complete) set of measurements to establish a family of positive, trace-preserving maps and the associated entanglement witnesses. These witnesses are also related to the class based on the CCNR criterion^46^. Also, they do not depend on the purity of measurement operators but on their number inside each POVM. It turns out that one can construct the same entanglement witness using different measurements. Finally, we provide examples of indecomposable witnesses that belong to our class. Such witnesses are very important because they can detect bound entangled states. However, no general method of construction exists, and it is generally hard to determine whether a given witness is decomposable or not. This is the reason why finding more examples of indecomposable entanglement witnesses is so important.

## Classes of symmetric measurements

Quantum measurements are represented by positive, operator-valued measures (POVMs), which are families of positive operators that sum up to the identity. For a given density operator $\rho$, the probability outcome associated with a POVM element $E_\alpha$ is $p_\alpha =\mathrm {Tr}(E_\alpha \rho )$. Recently, a general class of symmetric measurements has been introduced^39^. Let us summarize its definition and main properties.

### Definition 1

An (*N*, *M*)-POVM is a collection of *N* *d*-dimensional POVMs $\{E_{\alpha ,k};\,k=1,\ldots ,M\}$, $\alpha =1,\ldots ,N$, that satisfy the symmetry conditions

1 $$\begin{aligned} \begin{aligned} \mathrm {Tr}(E_{\alpha ,k})&=\frac{d}{M},\\ \mathrm {Tr}(E_{\alpha ,k}^2)&=x,\\ \mathrm {Tr}(E_{\alpha ,k}E_{\alpha ,\ell })&=\frac{d-Mx}{M(M-1)},\qquad \ell \ne k,\\ \mathrm {Tr}(E_{\alpha ,k}E_{\beta ,\ell })&=\frac{d}{M^2},\qquad \beta \ne \alpha , \end{aligned} \end{aligned}$$

with a free parameter *x* that belongs to the range

2 $$\begin{aligned} \frac{d}{M^2}<x\le \min \left\{ \frac{d^2}{M^2},\frac{d}{M}\right\} . \end{aligned}$$

If $x=d^2/M^2$, then the (*N*, *M*)-POVM describes projective measurements. Moreover, Eq. (2) implies that there are no projective (*N*, *M*)-POVMs for $M<d$. Symmetric measurements are informationally complete if and only if

3 $$\begin{aligned} N=\frac{d^2-1}{M-1}. \end{aligned}$$

Observe that if $d=2$, there are only two possible choices of admissible *M* and *N*: $(N=1,M=4)$ for the general symmetric, informationally complete (SIC) POVMs^22^ and $(N=3,M=2)$ for mutually unbiased measurements (MUMs)^23^. In this case, Definition 1 only provides a unified method to describe generalizations of SIC POVMs and mutually unbiased bases (MUBs). However, this changes for $d>2$.

### Proposition 1

In any dimension $d<\infty$, there exist at least four distinct classes of informationally complete (*N*, *M*)-POVMs depending on the choice of *M* and *N*:

(i) $M=d^2$ and $N=1$ (general SIC POVM),
(ii) $M=d$ and $N=d+1$ (MUMs),
(iii) $$M=2$$ and $$N=d^2-1$$,
(iv) $$M=d+2$$ and $$N=d-1$$.

Informationally complete (*N*, *M*)-POVMs can be constructed in arbitrary dimensions using orthonormal Hermitian operator bases $\{G_0={\mathbb {I}}_d/\sqrt{d},G_{\alpha ,k};\,\alpha =1,\ldots ,N,\,k=1,\ldots ,M-1\}$ with $\mathrm {Tr}G_{\alpha ,k}=0$. Namely,

4 $$\begin{aligned} E_{\alpha ,k}=\frac{1}{M} {\mathbb {I}}_d+tH_{\alpha ,k}, \end{aligned}$$

where

5 $$\begin{aligned} H_{\alpha ,k}=\left\{ \begin{aligned} & G_\alpha -\sqrt{M}(\sqrt{M}+1)G_{\alpha ,k},\quad k=1,\ldots ,M-1,\\&(\sqrt{M}+1)G_\alpha ,\qquad k=M, \end{aligned}\right. \end{aligned}$$

and $G_\alpha =\sum _{k=1}^{M-1}G_{\alpha ,k}$. The parameter *t* is related to *x* via

6 $$\begin{aligned} x=\frac{d}{M^2}+t^2(M-1)(\sqrt{M}+1)^2. \end{aligned}$$

The optimal value $x_\mathrm{opt}$, which is the greatest *x* such that $E_{\alpha ,k}\ge 0$, depends on the operator basis.

Symmetric measurements admit an important property that becomes useful later on.

### Proposition 2

For $L\le N$ and an arbitrary state $\rho$, the elements of an (*N*, *M*)-POVM satisfy

7 $$\begin{aligned} \sum _{\alpha =1}^L\sum _{k=1}^Mp_{\alpha ,k}^2\le \frac{L}{M} +\frac{(M^2x-d)[d\mathrm {Tr}(\rho ^2)-1]}{dM(M-1)}, \end{aligned}$$

where $$p_{\alpha ,k}=\mathrm {Tr}(E_{\alpha ,k}\rho )$$.

### Proof

We follow the method presented in ref.^33^. First, observe that $H_{\alpha ,k}$ are traceless operators that satisfy the relations

8 $$\begin{aligned} \begin{aligned} \mathrm {Tr}(H_{\alpha ,k}^2)&=(M-1)(\sqrt{M}+1)^2,\\ \mathrm {Tr}(H_{\alpha ,k}H_{\alpha ,\ell })&=-(\sqrt{M}+1)^2,\qquad \ell \ne k,\\ \mathrm {Tr}(H_{\alpha ,k}H_{\beta ,\ell })&=0,\qquad \beta \ne \alpha . \end{aligned} \end{aligned}$$

Now, similarly to $G_{\alpha ,k}$, $H_{\alpha ,k}$ span the space of traceless Hermitian operators. Therefore, any quantum state $\rho$ can be represented as

9 $$\begin{aligned} \rho =\frac{1}{d} {\mathbb {I}}_d+\sum _{\alpha =1}^N\sum _{k=1}^Mr_{\alpha ,k}H_{\alpha ,k} \end{aligned}$$

with real-valued parameters $r_{\alpha ,k}$. Next, we calculate

10 $$\begin{aligned} p_{\alpha ,k}=\mathrm {Tr}(\rho E_{\alpha ,k})=\frac{1}{M} + t\mathrm {Tr}(\rho H_{\alpha ,k})= \frac{1}{M} + t\sum _{\ell =1}^Mr_{\alpha ,k}\mathrm {Tr}(H_{\alpha ,k}H_{\alpha ,\ell }) =\frac{1}{M} +t(\sqrt{M}+1)^2(Mr_{\alpha ,k}-r_\alpha ), \end{aligned}$$

where $r_\alpha =\sum _{k=1}^Mr_{\alpha ,k}$. The sum of squares reads

11 $$\begin{aligned} \sum _{k=1}^Mp_{\alpha ,k}^2=\frac{1}{M}+t^2M(\sqrt{M}+1)^4\left( M\sum _{k=1}^M r_{\alpha ,k}^2-r_\alpha ^2\right) . \end{aligned}$$

Finally, we take the sum over $\alpha =1,\ldots ,L$ and get

12 $$\begin{aligned} \sum _{\alpha =1}^L\sum _{k=1}^Mp_{\alpha ,k}^2=\frac{L}{M}+t^2M(\sqrt{M}+1)^4\sum _{\alpha =1}^L\left( M\sum _{k=1}^M r_{\alpha ,k}^2-r_\alpha ^2\right) . \end{aligned}$$

It remains to notice that

13 $$\begin{aligned} \mathrm {Tr}(\rho ^2)-\frac{1}{d} =\sum _{\alpha =1}^N\sum _{k,\ell =1}^Mr_{\alpha ,k}r_{\alpha ,\ell }\mathrm {Tr}(H_{\alpha ,k}H_{\alpha ,\ell }) \ge \sum _{\alpha =1}^L\sum _{k,\ell =1}^Mr_{\alpha ,k}r_{\alpha ,\ell }\mathrm {Tr}(H_{\alpha ,k}H_{\alpha ,\ell }) =(\sqrt{M}+1)^2\left[ M\sum _{k=1}^Mr_{\alpha ,k}^2-r_\alpha ^2\right] . \end{aligned}$$

Applying this result in Eq. (12), we arrive at

14 $$\begin{aligned} \sum _{\alpha =1}^L\sum _{k=1}^Mp_{\alpha ,k}^2\le \frac{L}{M} +t^2M(\sqrt{M}+1)^2\left[ \mathrm {Tr}(\rho ^2)-\frac{1}{d} \right] =\frac{L}{M} +\frac{(M^2x-d)[d\mathrm {Tr}(\rho ^2)-1]}{dM(M-1)} \end{aligned}$$

after using the correspondence between *t* and *x* in eq. (6). $\square$

For $L=N$, the relation in Eq. (7) is an equality and it reproduces the result first obtained in ref.^39^,

15 $$\begin{aligned} \sum _{\alpha =1}^N\sum _{k=1}^Mp_{\alpha ,k}^2=\frac{d(M^2x-d)\mathrm {Tr}(\rho ^2)+d^3-M^2x}{dM(M-1)}. \end{aligned}$$

On the other hand, for pure states $\rho$, Eq. (7) reduces to

16 $$\begin{aligned} \sum _{\alpha =1}^L\sum _{k=1}^Mp_{\alpha ,k}^2\le \frac{L}{M} +\frac{(d-1)(M^2x-d)}{dM(M-1)}. \end{aligned}$$

## Positive maps and entanglement witnesses

Using any (*N*, *M*)-POVM (that is not necessarily informationally complete), define *N* trace-preserving maps

17 $$\begin{aligned} \Phi _\alpha [X]=\frac{M}{d} \sum _{k,\ell =1}^{M}{\mathcal {O}}^{(\alpha )}_{k\ell }E_{\alpha ,k}\mathrm {Tr}(XE_{\alpha ,\ell }), \end{aligned}$$

where ${\mathcal {O}}^{(\alpha )}=({\mathcal {O}}^{(\alpha )}_{k\ell })$ are orthogonal rotations that preserve the maximally mixed vector ${\mathbf {n}}_*=(1,\ldots ,1)/\sqrt{d}$.

### Proposition 3

The linear map

18 $$\begin{aligned} \Phi =\frac{1}{b}\left[ a\Phi _0+\sum _{\alpha =L+1}^N\Phi _\alpha -\sum _{\alpha =1}^L\Phi _\alpha \right] \end{aligned}$$

with $a=b-N+2L$ and $b=(d-1)M(x-y)/d$ is positive and trace-preserving. $\Phi _0$ is the maximally depolarizing channel.

### Proof

It suffices to prove that

19 $$\begin{aligned} \mathrm {Tr}(\Phi [P])^2\le \frac{1}{d-1} \end{aligned}$$

holds for every rank-1 projectors *P*^8^. For simplicity, introduce the map $\widetilde{\Phi }=b\Phi$, and then calculate

20 $$\begin{aligned} \begin{aligned} \mathrm {Tr}(\widetilde{\Phi }[P])^2=\mathrm {Tr}\Bigg \{&a^2\Phi _0[P]^2+\sum _{\alpha ,\beta =1}^L \Phi _\alpha [P]\Phi _\beta [P]+\sum _{\alpha ,\beta =L+1}^N\Phi _\alpha [P]\Phi _\beta [P] +2a\sum _{\alpha =L+1}^N\Phi _0[P]\Phi _\alpha [P]\\&-2a\sum _{\alpha =1}^L\Phi _0[P]\Phi _\alpha [P] -2\sum _{\alpha =L+1}^N\sum _{\beta =1}^L\Phi _\alpha [P]\Phi _\beta [P]\Bigg \}. \end{aligned} \end{aligned}$$

First, let us simplify the terms in the brackets using the properties of (*N*, *M*)-POVMs and the orthogonal rotation matrices,

21 $$\begin{aligned} \sum _{k=1}^M{\mathcal {O}}^{(\alpha )}_{k\ell }=\sum _{\ell =1}^M{\mathcal {O}}^{(\alpha )}_{k\ell }=1,\qquad \sum _{k=1}^M{\mathcal {O}}^{(\alpha )}_{k\ell }{\mathcal {O}}^{(\alpha )}_{km}=\delta _{\ell m}. \end{aligned}$$

One has

22 $$\begin{aligned} \mathrm {Tr}(\Phi _0[P]^2)=\mathrm {Tr}(\Phi _0[P]\Phi _\alpha [P])=\mathrm {Tr}(\Phi _\alpha [P]\Phi _\beta [P])=\frac{1}{d},\quad \alpha \ne \beta , \end{aligned}$$

as well as

23 $$\begin{aligned} \mathrm {Tr}(\Phi _\alpha [P]^2)=\frac{M}{d^2(M-1)}\left\{ d-Mx+(M^2x-d) \sum _{k=1}^M\left[ \mathrm {Tr}(PE_{\alpha ,k})\right] ^2\right\} . \end{aligned}$$

Now, Eq. (20) can be rewritten as

24 $$\begin{aligned} \begin{aligned} \mathrm {Tr}(\widetilde{\Phi }[P])^2=&\,\frac{1}{d} \Big [a^2+(N-L)(N-L-1)+L(L-1)+2a(N-L)-2aL -2L(N-L)\Big ]\\ {}&+\frac{M}{d^2(M-1)}\left\{ N(d-Mx)+(M^2x-d) \sum _{\alpha =1}^N\sum _{k=1}^M\left[ \mathrm {Tr}(PE_{\alpha ,k})\right] ^2\right\} \\ =&\frac{1}{d} \Big [(a+N-2L)^2-N\Big ]+\frac{M}{d^2(M-1)}\left\{ N(d-Mx)+(M^2x-d) \sum _{\alpha =1}^N\sum _{k=1}^M\left[ \mathrm {Tr}(PE_{\alpha ,k})\right] ^2\right\} . \end{aligned} \end{aligned}$$

Finally, Eq. (7) from Proposition 2 and the definition of *a* allow us to write

25 $$\begin{aligned} \begin{aligned} \mathrm {Tr}(\widetilde{\Phi }[P])^2\le \frac{b^2-N}{d} +\frac{MN(d-Mx)}{d^2(M-1)}+\frac{M(M^2x-d)}{d^2(M-1)}\left[ \frac{N}{M} +\frac{(d-1)(M^2x-d)}{dM(M-1)}\right] = \frac{b^2}{d-1}, \end{aligned} \end{aligned}$$

which finally proves the validity of condition (19). $\square$

Note that, in the above proof, the conditions $E_{\alpha ,k}\ge 0$ were not used to show the positivity of $\Phi$. Therefore, this map can actually be defined using $E_{\alpha ,k}\ngeq 0$, which makes the construction more general than initially assumed.

Positive maps are used in the theory of quantum entanglement to detect entangled states; that is, states that are not separable. Positive but not completely positive maps $\Phi$ define entanglement witnesses *W* through the Choi-Jamiołkowski isomorphism,

26 $$\begin{aligned} W=\sum _{k,\ell =0}^{d-1}|k{\rangle }{\langle }\ell |\otimes \Phi [|k{\rangle }{\langle }\ell |], \end{aligned}$$

where $|k\>$ is an orthonormal basis in ${\mathbb {C}}^d$. In particular, the positive map $\Phi$ from Proposition 3 gives rise to

27 $$\begin{aligned} W=\frac{1}{b}\left( \frac{a}{d} {\mathbb {I}}_{d^2} +\sum _{\alpha =L+1}^NK_\alpha -\sum _{\alpha =1}^LK_\alpha \right) , \end{aligned}$$

where

28 $$\begin{aligned} K_\alpha =\frac{M}{d} \sum _{k,\ell =1}^M{\mathcal {O}}_{k\ell }^{(\alpha )} \overline{E}_{\alpha ,\ell }\otimes E_{\alpha ,k}, \end{aligned}$$

and $\overline{E}_{\alpha ,\ell }$ denotes the complex conjugate of ${E}_{\alpha ,\ell }$. In any dimension *d*, one can always construct informationally complete (*N*, *M*)-POVMs by using an orthonormal basis $\{{\mathbb {I}}_d/\sqrt{d},G_{\alpha ,k}\}$ of traceless Hermitian operators $G_{\alpha ,k}$. Then, the entanglement witness can be expressed in terms of the operator basis,

29 $$\begin{aligned} \widetilde{W}=\frac{b}{t^2} W = \frac{d-1}{d^2}M^2(\sqrt{M}+1)^2 {\mathbb {I}}_{d^2}+\sum _{\alpha =L+1}^NJ_\alpha -\sum _{\alpha =1}^LJ_\alpha , \end{aligned}$$

where

30 $$\begin{aligned} J_\alpha =\frac{M}{d} \sum _{k,\ell =1}^{M}{\mathcal {O}}_{kl}^{(\alpha )} \overline{H}_{\alpha ,\ell }\otimes H_{\alpha ,k}. \end{aligned}$$

Note that this witness does not depend on the parameter *x*. This is because *x* characterizes symmetric measurements rather than operator bases. However, the dependence on the choice of symmetric measurements manifests itself through the number *M* of measurement operators inside a single POVM. The greater the value of *M*, the greater *L* can be (the more can be subtracted).

Going a step further, $\widetilde{W}$ can be directly written in terms of the operator basis elements $G_{\alpha ,k}$. Using eqs. (5) and (30), it is straightforward to find

31 $$\begin{aligned} J_\alpha =\frac{M}{d} \sum _{k,\ell =1}^{M-1}{\mathcal {Q}}_{kl}^{(\alpha )}\overline{G}_{\alpha ,\ell } \otimes G_{\alpha ,k} \end{aligned}$$

with

32 $$\begin{aligned} {\mathcal {Q}}_{k\ell }^{(\alpha )} = M({\mathcal {O}}_{MM}^{(\alpha )}-1) + M(\sqrt{M}+1)^2 {\mathcal {O}}_{k\ell }^{(\alpha )} - M(\sqrt{M}+1) ({\mathcal {O}}_{M\ell }^{(\alpha )}+{\mathcal {O}}_{kM}^{(\alpha )}). \end{aligned}$$

Such ${\mathcal {Q}}^{(\alpha )}$ are rescaled orthogonal matrices since they satisfy

33 $$\begin{aligned} {\mathcal {Q}}^{(\alpha )T}{\mathcal {Q}}^{(\alpha )} ={\mathcal {Q}}^{(\alpha )}{\mathcal {Q}}^{(\alpha )T}=M^2(\sqrt{M}+1)^4{\mathbb {I}}_{M-1}. \end{aligned}$$

For the special case with ${\mathcal {O}}^{(\alpha )}={\mathbb {I}}_M$, one has

34 $$\begin{aligned} {\mathcal {Q}}_{k\ell }^{(\alpha )} = M(\sqrt{M}+1)^2 \delta _{k\ell }. \end{aligned}$$

Now, let us show how $\widetilde{W}$ from eq. (29) relates to a well-known class of entanglement witnesses. For now, assume that the (*N*, *M*)-POVM is informationally complete and $L=N$. In this case, the associated witness reads

35 $$\begin{aligned} \widetilde{W}=\frac{M^2}{d}(\sqrt{M}+1)^2\left[ {\mathbb {I}}_{d^2}- G_0\otimes G_0-\frac{d}{M^2(\sqrt{M}+1)^2}\sum _{\alpha =1}^{N}J_\alpha \right] , \end{aligned}$$

where $G_0={\mathbb {I}}_d/\sqrt{d}$. A simple relabelling of indices $(\alpha ,k)\longmapsto \mu$ allows us to write

36 $$\begin{aligned} \widetilde{W}^\prime =\frac{d\widetilde{W}}{M^2(\sqrt{M}+1)^2}={\mathbb {I}}_{d^2} -\sum _{\mu ,\nu =0}^{d^2-1}Q_{\mu \nu } G_\mu ^T\otimes G_\nu , \end{aligned}$$

where we introduced the block-diagonal orthogonal matrix

37 $$\begin{aligned} Q=\frac{1}{M(\sqrt{M}+1)^2} \left[ \begin{array}{c c c c c} M(\sqrt{M}+1)^2 &{} &{} &{} &{} \\ &{} {\mathcal {Q}}^{(1)T} &{} &{} &{} \\ &{} &{} {\mathcal {Q}}^{(2)T} &{} &{} \\ &{} &{} &{} \ddots &{} \\ &{} &{} &{} &{} {\mathcal {Q}}^{(N)T} \end{array}\right] . \end{aligned}$$

Now, it becomes evident that entanglement witnesses $\widetilde{W}$ constructed from symmetric measurements are a part of a larger class

38 $$\begin{aligned} W^\prime ={\mathbb {I}}_{d^2}-\sum _{\mu ,\nu =0}^{d^2-1}Q_{\mu \nu } G_\mu ^T\otimes G_\nu , \end{aligned}$$

which is related to the CCNR criterion^46^. In the above formula, $G_\mu$ are the elements of an arbitrary orthonormal Hermitian basis and $Q_{\mu \nu }$ is any orthogonal matrix ($Q^TQ={\mathbb {I}}_{d^2}$). However, it is enough that $Q^TQ\le {\mathbb {I}}_{d^2}$. Hence, our discussion also holds true for any (*N*, *M*)-POVMs and $L\le N$, provided that the rotation matrices ${\mathcal {O}}^{(\alpha )}$ get replaced with $\widetilde{{\mathcal {O}}}^{(\alpha )}$ that are allowed to change the sign of ${\mathbf {n}}_*$ ($\widetilde{{\mathcal {O}}}^{(\alpha )}{\mathbf {n}}_*=\pm {\mathbf {n}}_*$) or vanish ($\widetilde{{\mathcal {O}}}^{(\alpha )}={\mathbb {O}}_M$).

### Example 1

Take any informationally complete (*N*, *M*)-POVM. From eq. (34), it follows that fixing all ${\mathcal {O}}^{(\alpha )}={\mathbb {I}}_M$ and $L=N$ always results in $Q={\mathbb {I}}_{d^2}$. The corresponding entanglement witness has the form

39 $$\begin{aligned} \widetilde{W}^\prime ={\mathbb {I}}_{d^2}-\sum _{\mu =0}^{d^2-1} G_\mu ^T\otimes G_\mu . \end{aligned}$$

Therefore, it is possible to produce the same witnesses $\widetilde{W}^\prime$ using different (*N*, *M*)-POVMs, provided that they arise from the same Hermitian orthonormal basis. In particular, for the Gell-Mann matrices, one recovers the reduction map $\widetilde{W}^\prime ={\mathbb {I}}_{d^2}-dP_+$^7,40^.

### Example 2

If $M=2$, then the only admissible choices for the rotation matrices are ${\mathcal {O}}^{(\alpha )}={\mathbb {I}}_2$ or ${\mathcal {O}}^{(\alpha )}=\sigma _1$. This means that all witnesses constructed from (*N*, 2)-POVMs have the form

40 $$\begin{aligned} \widetilde{W}^\prime = {\mathbb {I}}_{d^2}+\sum _{\alpha =L+1}^N G_\alpha ^T\otimes G_\alpha -\sum _{\alpha =0}^L G_\alpha ^T\otimes G_\alpha , \end{aligned}$$

where $$N\le d^2-1$$.

## Indecomposable witnesses in $d=3$

Let us consider composite quantum systems with the underlying Hilbert space ${\mathcal {H}}\simeq {\mathbb {C}}^3\otimes {\mathbb {C}}^3$, which corresponds to fixing $d=3$. These are the lowest dimensional bipartite systems where the Peres-Horodecki PPT criterion is only necessary for separability. Therefore, more sophisticated methods of entanglement detection are needed. An important class of entanglement witnesses are indecomposable witnesses, which detect more entanglement than transposition *T*. Recall that a witness is decomposable if and only if it can be represented as $W=A+B^\Gamma$ with *A*, *B* being positive operators and $\Gamma =\mathbbm {1}\otimes T$ denoting a partial transposition. Otherwise, it is indecomposable. Construction of indecomposable witnesses is hard but rewarding, as they can detect quantum states with positive partial transposition (PPT states).

Below, we present several examples of indecomposable witnesses that can be obtained from symmetric measurements. To show indecomposability of a witness, it is enough to find a single PPT state that it detects. This state is then known to be PPT entangled. Hence, in what follows, we postulate the form of $\rho$ analogical to $\widetilde{W}$, and then look for a relatively simple set of parameters for which $\rho ^\Gamma \ge 0$ and $\mathrm {Tr}(\widetilde{W}\rho )<0$. A proof that the proposed states are indeed PPT can be found in Appendix C.

### Example 3

First, consider a full set of $N=4$ mutually unbiased measurements ($M=3$) constructed from the Gell-Mann matrices (see Appendix A). Note that for this choice of an orthonormal basis, the parameter $x=5/9<x_\mathrm{opt}=1$ is not optimal. Now, for $L=3$ and the permutation matrices

41 $$\begin{aligned} {\mathcal {O}}^{(\alpha )}=\begin{pmatrix} 0 &{} 1 &{} 0 \\ 0 &{} 0 &{} 1 \\ 1 &{} 0 &{} 0 \end{pmatrix}, \end{aligned}$$

the associated entanglement witness

42 $$\begin{aligned} \widetilde{W}_1=\frac{1}{6} \left[ \begin{array}{c c c|c c c|c c c} 2 &{} \cdot &{} \cdot &{} \cdot &{} 3(1-i\sqrt{3}) &{} \cdot &{} \cdot &{} \cdot &{} 3(1-i\sqrt{3}) \\ \cdot &{} 2 &{} \cdot &{} \cdot &{} \cdot &{} \cdot &{} \cdot &{} \cdot &{} \cdot \\ \cdot &{} \cdot &{} 8 &{} \cdot &{} \cdot &{} \cdot &{} \cdot &{} \cdot &{} \cdot \\ \hline \cdot &{} \cdot &{} \cdot &{} 8 &{} \cdot &{} \cdot &{} \cdot &{} \cdot &{} \cdot \\ 3(1+i\sqrt{3}) &{} \cdot &{} \cdot &{} \cdot &{} 2 &{} \cdot &{} \cdot &{} \cdot &{} 3(1-i\sqrt{3}) \\ \cdot &{} \cdot &{} \cdot &{} \cdot &{} \cdot &{} 2 &{} \cdot &{} \cdot &{} \cdot \\ \hline \cdot &{} \cdot &{} \cdot &{} \cdot &{} \cdot &{} \cdot &{} 2 &{} \cdot &{} \cdot \\ \cdot &{} \cdot &{} \cdot &{} \cdot &{} \cdot &{} \cdot &{} \cdot &{} 8 &{} \cdot \\ 3(1+i\sqrt{3}) &{} \cdot &{} \cdot &{} \cdot &{} 3(1+i\sqrt{3}) &{} \cdot &{} \cdot &{} \cdot &{} 2 \end{array}\right] \end{aligned}$$

is indecomposable, as it detects the PPT state

43 $$\begin{aligned} \rho _1=\frac{1}{579} \left[ \begin{array}{c c c|c c c|c c c} 125 &{} \cdot &{} \cdot &{} \cdot &{} -5(5-12i) &{} \cdot &{} \cdot &{} \cdot &{} -5(5-12i) \\ \cdot &{} 125 &{} \cdot &{} \cdot &{} \cdot &{} \cdot &{} \cdot &{} \cdot &{} \cdot \\ \cdot &{} \cdot &{} 34 &{} \cdot &{} \cdot &{} \cdot &{} \cdot &{} \cdot &{} \cdot \\ \hline \cdot &{} \cdot &{} \cdot &{} 34 &{} \cdot &{} \cdot &{} \cdot &{} \cdot &{} \cdot \\ -5(5+12i) &{} \cdot &{} \cdot &{} \cdot &{} 125 &{} \cdot &{} \cdot &{} \cdot &{} -5(5-12i) \\ \cdot &{} \cdot &{} \cdot &{} \cdot &{} \cdot &{} 125 &{} \cdot &{} \cdot &{} \cdot \\ \hline \cdot &{} \cdot &{} \cdot &{} \cdot &{} \cdot &{} \cdot &{} 125 &{} \cdot &{} \cdot \\ \cdot &{} \cdot &{} \cdot &{} \cdot &{} \cdot &{} \cdot &{} \cdot &{} 34 &{} \cdot \\ -5(5+12i) &{} \cdot &{} \cdot &{} \cdot &{} -5(5+12i) &{} \cdot &{} \cdot &{} \cdot &{} 125 \end{array}\right] . \end{aligned}$$

Therefore, we show that *x* does not have to be optimal in order to produce indecomposable witnesses, which was inconclusive in ref.^40^. Also, note that the state $\rho _1$ is not detected if the witness in Example 3 is analogously constructed from the MUBs instead. This means that an entanglement witness constructed from measurements with $x<x_\mathrm{opt}$ can detect entanglement undetectable with $x=x_\mathrm{opt}$.

### Example 4

Now, we take the (2, *N*)-POVM constructed from the orthonormal Hermitian basis presented in Appendix B, for which $x=3(5-2\sqrt{3})/4\simeq 1.15<x_\mathrm{opt}=3/2$ is not optimal. Assume that all the rotation matrices are ${\mathcal {O}}^{(\alpha )}={\mathbb {I}}_2$. An indecomposable witness follows for a non-maximal number of $N=7$ POVMs and $L=3$. Indeed, one has

44 $$\begin{aligned} \widetilde{W}_2=\frac{1}{6} \left[ \begin{array}{c c c|c c c|c c c} 2+\sqrt{3} &{} \cdot &{} \cdot &{} \cdot &{} 2 &{} \cdot &{} \cdot &{} \cdot &{} 2 \\ \cdot &{} 5 &{} \cdot &{} \cdot &{} \cdot &{} -4 &{} -4 &{} \cdot &{} \cdot \\ \cdot &{} \cdot &{} 5-\sqrt{3} &{} -4 &{} \cdot &{} \cdot &{} \cdot &{} -4 &{} \cdot \\ \hline \cdot &{} \cdot &{} -4 &{} 5 &{} \cdot &{} \cdot &{} \cdot &{} -4 &{} \cdot \\ 2 &{} \cdot &{} \cdot &{} \cdot &{} 2-\sqrt{3} &{} \cdot &{} \cdot &{} \cdot &{} 2 \\ \cdot &{} -4 &{} \cdot &{} \cdot &{} \cdot &{} 5+\sqrt{3} &{} -4 &{} \cdot &{} \cdot \\ \hline \cdot &{} -4 &{} \cdot &{} \cdot &{} \cdot &{} -4 &{} 5-\sqrt{3} &{} \cdot &{} \cdot \\ \cdot &{} \cdot &{} -4 &{} -4 &{} \cdot &{} \cdot &{} \cdot &{} 5+\sqrt{3} &{} \cdot \\ 2 &{} \cdot &{} \cdot &{} \cdot &{} 2 &{} \cdot &{} \cdot &{} \cdot &{} 2 \end{array}\right] , \end{aligned}$$

which detects the PPT state

45 $$\begin{aligned} \rho _2=\frac{1}{21} \left[ \begin{array}{c c c|c c c|c c c} 3 &{} \cdot &{} \cdot &{} \cdot &{} 1 &{} \cdot &{} \cdot &{} \cdot &{} 1 \\ \cdot &{} 2 &{} \cdot &{} \cdot &{} \cdot &{} 2 &{} 2 &{} \cdot &{} \cdot \\ \cdot &{} \cdot &{} 2 &{} 2 &{} \cdot &{} \cdot &{} \cdot &{} 2 &{} \cdot \\ \hline \cdot &{} \cdot &{} 2 &{} 2 &{} \cdot &{} \cdot &{} \cdot &{} 2 &{} \cdot \\ 1 &{} \cdot &{} \cdot &{} \cdot &{} 3 &{} \cdot &{} \cdot &{} \cdot &{} 1 \\ \cdot &{} 2 &{} \cdot &{} \cdot &{} \cdot &{} 2 &{} 2 &{} \cdot &{} \cdot \\ \hline \cdot &{} 2 &{} \cdot &{} \cdot &{} \cdot &{} 2 &{} 2 &{} \cdot &{} \cdot \\ \cdot &{} \cdot &{} 2 &{} 2 &{} \cdot &{} \cdot &{} \cdot &{} 2 &{} \cdot \\ 1 &{} \cdot &{} \cdot &{} \cdot &{} 1 &{} \cdot &{} \cdot &{} \cdot &{} 3 \end{array}\right] . \end{aligned}$$

Hence, the full set of $N=9$ POVMs is not needed to construct indecomposable witnesses.

### Example 5

Let us analyze an entanglement witness defined using the (5, *N*)-POVM with the associated Hermitian operator basis of the Gell-Mann matrices (see Appendix A). Also for $M=5$, the Gell-Mann basis produces symmetric measurements with the parameter $x\simeq 0.183<x_\mathrm{opt}=9/25$ that is not optimal. In this example, we assume that only subtractions are present ($L=N$) and that the (*M*, *N*)-POVM is not informationally complete ($N=1$). Now, if we take the permutation matrix

46 $$\begin{aligned} {\mathcal {O}}^{(1)}=\begin{pmatrix} 0 &{} 0 &{} 0 &{} 0 &{} 1 \\ 1 &{} 0 &{} 0 &{} 0 &{} 0 \\ 0 &{} 1 &{} 0 &{} 0 &{} 0 \\ 0 &{} 0 &{} 1 &{} 0 &{} 0 \\ 0 &{} 0 &{} 0 &{} 1 &{} 0 \end{pmatrix}, \end{aligned}$$

the decomposability property of the resulting witness $\widetilde{W}_3$ is inconclusive. However, by subtracting $5(1+\sqrt{5})^2$ times more of the term for $\alpha =1$, one obtains

47 $$\begin{aligned} \widetilde{W}^\prime _3=\frac{1}{6} \left[ \begin{array}{c c c|c c c|c c c} 4 &{} \cdot &{} \cdot &{} \cdot &{} B^*&{} C &{} \cdot &{} D^*&{} B^*\\ \cdot &{} 4 &{} \cdot &{} A^*&{} \cdot &{} \cdot &{} -30i &{} \cdot &{} \cdot \\ \cdot &{} \cdot &{} 4 &{} 30i &{} \cdot &{} \cdot &{} -A&{} \cdot &{} \cdot \\ \hline \cdot &{} A &{} -30i &{} 4 &{} \cdot &{} \cdot &{} \cdot &{} \cdot &{} \cdot \\ B &{} \cdot &{} \cdot &{} \cdot &{} 4 &{} \cdot &{} \cdot &{} \cdot &{} \cdot \\ C &{} \cdot &{} \cdot &{} \cdot &{} \cdot &{} 4 &{} \cdot &{} \cdot &{} \cdot \\ \hline \cdot &{} 30i &{} -A^*&{} \cdot &{} \cdot &{} \cdot &{} 4 &{} \cdot &{} \cdot \\ D &{} \cdot &{} \cdot &{} \cdot &{} \cdot &{} \cdot &{} \cdot &{} 4 &{} \cdot \\ B &{} \cdot &{} \cdot &{} \cdot &{} \cdot &{} \cdot &{} \cdot &{} \cdot &{} 4 \end{array}\right] , \end{aligned}$$

where

48 $$\begin{aligned} A&=15(1-i)(2-i+\sqrt{5}), \end{aligned}$$

49 $$\begin{aligned} B&=15(1-i)(2+i+\sqrt{5}),\end{aligned}$$

50 $$\begin{aligned} C&=-30\sqrt{5}(2+\sqrt{5}),\end{aligned}$$

51 $$\begin{aligned} D&=30(1-2i)(2+\sqrt{5}). \end{aligned}$$

This entanglement witness is indecomposable, and it detects the PPT state

52 $$\begin{aligned} \rho _3=\frac{1}{90} \left[ \begin{array}{c c c|c c c|c c c} 10 &{} \cdot &{} \cdot &{} \cdot &{} \cdot &{} \cdot &{} \cdot &{} \cdot &{} \cdot \\ \cdot &{} 10 &{} \cdot &{} 3-6i &{} \cdot &{} \cdot &{} \cdot &{} \cdot &{} \cdot \\ \cdot &{} \cdot &{} 10 &{} \cdot &{} \cdot &{} \cdot &{} -3-6i &{} \cdot &{} \cdot \\ \hline \cdot &{} 3+6i &{} \cdot &{} 10 &{} \cdot &{} \cdot &{} \cdot &{} \cdot &{} \cdot \\ \cdot &{} \cdot &{} \cdot &{} \cdot &{} 10 &{} \cdot &{} \cdot &{} \cdot &{} \cdot \\ \cdot &{} \cdot &{} \cdot &{} \cdot &{} \cdot &{} 10 &{} \cdot &{} \cdot &{} \cdot \\ \hline \cdot &{} \cdot &{} -3+6i &{} \cdot &{} \cdot &{} \cdot &{} 10 &{} \cdot &{} \cdot \\ \cdot &{} \cdot &{} \cdot &{} \cdot &{} \cdot &{} \cdot &{} \cdot &{} 10 &{} \cdot \\ \cdot &{} \cdot &{} \cdot &{} \cdot &{} \cdot &{} \cdot &{} \cdot &{} \cdot &{} 10 \end{array}\right] \end{aligned}$$

that was undetectable by the previous (not optimal) witness $\widetilde{W}_3$.

Recall that the positivity conditions for $\Phi$ in Proposition 3 are only sufficient. Therefore, it is not surprising that we found an entanglement witness $\widetilde{W}^\prime$ which does not fall into the same class as $\widetilde{W}$ defined by such $\Phi$.

## Indecomposable witness in $d=4$

Our construction allows for considering higher dimensional quantum systems. Let us set $d=4$, which is equivalent to taking a composite system with the Hilbert space ${\mathcal {H}}\simeq {\mathbb {C}}^4\otimes {\mathbb {C}}^4$. For $d=4$, it was shown that there exists an optimal informationally complete (*N*, *M*)-POVM with $N=15$, $M=2$, and $x=x_\mathrm{opt}=2$ such whose measurement operators are rank-2 projectors^39^. They were constructed from the Hermitian basis that consists in a normalized tensor product of the Pauli matrices; that is, $G_{\alpha \beta }=\sigma _\alpha \otimes \sigma _\beta /2$, where $\alpha ,\beta =0,1,2,3$. In what follows, we use $G_{\alpha \beta }$ to define an indecomposable entanglement witness. For $M=2$, a general form of an entanglement witness is given in Example 2. Hence, taking $N=15$ and $L=12$, we construct the witness

53 $$\begin{aligned} \widetilde{W}_4={\mathbb {I}}_{16}+\sum _{\beta =1}^3\overline{G}_{0\beta }\otimes {G}_{0\beta }-G_{00}\otimes {G}_{00}-\sum _{\alpha =1}^3\sum _{\beta =0}^3 \overline{G}_{\alpha \beta }\otimes {G}_{\alpha \beta }, \end{aligned}$$

whose matrix representation reads

54 $$\begin{aligned} \widetilde{W}_4=\frac{1}{2} \left[ \begin{array}{c c c c|c c c c|c c c c|c c c c} 2 &{} \cdot &{} \cdot &{} \cdot &{} \cdot &{} 1 &{} \cdot &{} \cdot &{} \cdot &{} \cdot &{} -3 &{} \cdot &{} \cdot &{} \cdot &{} \cdot &{} -3 \\ \cdot &{} 1 &{} \cdot &{} \cdot &{} \cdot &{} \cdot &{} \cdot &{} \cdot &{} \cdot &{} \cdot &{} \cdot &{} \cdot &{} \cdot &{} \cdot &{} \cdot &{} \cdot \\ \cdot &{} \cdot &{} 2 &{} \cdot &{} \cdot &{} \cdot &{} \cdot &{} 1 &{} -3 &{} \cdot &{} \cdot &{} \cdot &{} \cdot &{} -3 &{} \cdot &{} \cdot \\ \cdot &{} \cdot &{} \cdot &{} 1 &{} \cdot &{} \cdot &{} \cdot &{} \cdot &{} \cdot &{} \cdot &{} \cdot &{} \cdot &{} \cdot &{} \cdot &{} \cdot &{} \cdot \\ \hline \cdot &{} \cdot &{} \cdot &{} \cdot &{} 1 &{} \cdot &{} \cdot &{} \cdot &{} \cdot &{} \cdot &{} \cdot &{} \cdot &{} \cdot &{} \cdot &{} \cdot &{} \cdot \\ 1 &{} \cdot &{} \cdot &{} \cdot &{} \cdot &{} 2 &{} \cdot &{} \cdot &{} \cdot &{} \cdot &{} -3 &{} \cdot &{} \cdot &{} \cdot &{} \cdot &{} -3 \\ \cdot &{} \cdot &{} \cdot &{} \cdot &{} \cdot &{} \cdot &{} 1 &{} \cdot &{} \cdot &{} \cdot &{} \cdot &{} \cdot &{} \cdot &{} \cdot &{} \cdot &{} \cdot \\ \cdot &{} \cdot &{} 1 &{} \cdot &{} \cdot &{} \cdot &{} \cdot &{} 2 &{} -3 &{} \cdot &{} \cdot &{} \cdot &{} \cdot &{} -3 &{} \cdot &{} \cdot \\ \hline \cdot &{} \cdot &{} -3 &{} \cdot &{} \cdot &{} \cdot &{} \cdot &{} -3 &{} 2 &{} \cdot &{} \cdot &{} \cdot &{} \cdot &{} 1 &{} \cdot &{} \cdot \\ \cdot &{} \cdot &{} \cdot &{} \cdot &{} \cdot &{} \cdot &{} \cdot &{} \cdot &{} \cdot &{} 1 &{} \cdot &{} \cdot &{} \cdot &{} \cdot &{} \cdot &{} \cdot \\ -3 &{} \cdot &{} \cdot &{} \cdot &{} \cdot &{} -3 &{} \cdot &{} \cdot &{} \cdot &{} \cdot &{} 2 &{} \cdot &{} \cdot &{} \cdot &{} \cdot &{} 1 \\ \cdot &{} \cdot &{} \cdot &{} \cdot &{} \cdot &{} \cdot &{} \cdot &{} \cdot &{} \cdot &{} \cdot &{} \cdot &{} 1 &{} \cdot &{} \cdot &{} \cdot &{} \cdot \\ \hline \cdot &{} \cdot &{} \cdot &{} \cdot &{} \cdot &{} \cdot &{} \cdot &{} \cdot &{} \cdot &{} \cdot &{} \cdot &{} \cdot &{} 1 &{} \cdot &{} \cdot &{} \cdot \\ \cdot &{} \cdot &{} -3 &{} \cdot &{} \cdot &{} \cdot &{} \cdot &{} -3 &{} 1 &{} \cdot &{} \cdot &{} \cdot &{} \cdot &{} 2 &{} \cdot &{} \cdot \\ \cdot &{} \cdot &{} \cdot &{} \cdot &{} \cdot &{} \cdot &{} \cdot &{} \cdot &{} \cdot &{} \cdot &{} \cdot &{} \cdot &{} \cdot &{} \cdot &{} 1 &{} \cdot \\ -3 &{} \cdot &{} \cdot &{} \cdot &{} \cdot &{} -3 &{} \cdot &{} \cdot &{} \cdot &{} \cdot &{} 1 &{} \cdot &{} \cdot &{} \cdot &{} \cdot &{} 2 \end{array}\right] . \end{aligned}$$

This witness is indecomposable, as it detects the 2-parameter family of PPT entangled states

55 $$\begin{aligned} \rho _{qp}=\frac{1}{8(q+4)}\left[ \begin{array}{c c c c|c c c c|c c c c|c c c c} q+2 &{} \cdot &{} \cdot &{} \cdot &{} \cdot &{} q &{} \cdot &{} \cdot &{} \cdot &{} \cdot &{} p &{} \cdot &{} \cdot &{} \cdot &{} \cdot &{} p \\ \cdot &{} 2 &{} \cdot &{} \cdot &{} \cdot &{} \cdot &{} \cdot &{} \cdot &{} \cdot &{} \cdot &{} \cdot &{} \cdot &{} \cdot &{} \cdot &{} \cdot &{} \cdot \\ \cdot &{} \cdot &{} q+2 &{} \cdot &{} \cdot &{} \cdot &{} \cdot &{} q &{} p &{} \cdot &{} \cdot &{} \cdot &{} \cdot &{} p &{} \cdot &{} \cdot \\ \cdot &{} \cdot &{} \cdot &{} 2 &{} \cdot &{} \cdot &{} \cdot &{} \cdot &{} \cdot &{} \cdot &{} \cdot &{} \cdot &{} \cdot &{} \cdot &{} \cdot &{} \cdot \\ \hline \cdot &{} \cdot &{} \cdot &{} \cdot &{} 2 &{} \cdot &{} \cdot &{} \cdot &{} \cdot &{} \cdot &{} \cdot &{} \cdot &{} \cdot &{} \cdot &{} \cdot &{} \cdot \\ q &{} \cdot &{} \cdot &{} \cdot &{} \cdot &{} q+2 &{} \cdot &{} \cdot &{} \cdot &{} \cdot &{} p &{} \cdot &{} \cdot &{} \cdot &{} \cdot &{} p \\ \cdot &{} \cdot &{} \cdot &{} \cdot &{} \cdot &{} \cdot &{} 2 &{} \cdot &{} \cdot &{} \cdot &{} \cdot &{} \cdot &{} \cdot &{} \cdot &{} \cdot &{} \cdot \\ \cdot &{} \cdot &{} q &{} \cdot &{} \cdot &{} \cdot &{} \cdot &{} q+2 &{} p &{} \cdot &{} \cdot &{} \cdot &{} \cdot &{} p &{} \cdot &{} \cdot \\ \hline \cdot &{} \cdot &{} p &{} \cdot &{} \cdot &{} \cdot &{} \cdot &{} p &{} q+2 &{} \cdot &{} \cdot &{} \cdot &{} \cdot &{} q &{} \cdot &{} \cdot \\ \cdot &{} \cdot &{} \cdot &{} \cdot &{} \cdot &{} \cdot &{} \cdot &{} \cdot &{} \cdot &{} 2 &{} \cdot &{} \cdot &{} \cdot &{} \cdot &{} \cdot &{} \cdot \\ p &{} \cdot &{} \cdot &{} \cdot &{} \cdot &{} p &{} \cdot &{} \cdot &{} \cdot &{} \cdot &{} q+2 &{} \cdot &{} \cdot &{} \cdot &{} \cdot &{} q \\ \cdot &{} \cdot &{} \cdot &{} \cdot &{} \cdot &{} \cdot &{} \cdot &{} \cdot &{} \cdot &{} \cdot &{} \cdot &{} 2 &{} \cdot &{} \cdot &{} \cdot &{} \cdot \\ \hline \cdot &{} \cdot &{} \cdot &{} \cdot &{} \cdot &{} \cdot &{} \cdot &{} \cdot &{} \cdot &{} \cdot &{} \cdot &{} \cdot &{} 2 &{} \cdot &{} \cdot &{} \cdot \\ \cdot &{} \cdot &{} p &{} \cdot &{} \cdot &{} \cdot &{} \cdot &{} p &{} q &{} \cdot &{} \cdot &{} \cdot &{} \cdot &{} q+2 &{} \cdot &{} \cdot \\ \cdot &{} \cdot &{} \cdot &{} \cdot &{} \cdot &{} \cdot &{} \cdot &{} \cdot &{} \cdot &{} \cdot &{} \cdot &{} \cdot &{} \cdot &{} \cdot &{} 2 &{} \cdot \\ p &{} \cdot &{} \cdot &{} \cdot &{} \cdot &{} p &{} \cdot &{} \cdot &{} \cdot &{} \cdot &{} q &{} \cdot &{} \cdot &{} \cdot &{} \cdot &{} q+2 \end{array}\right] , \end{aligned}$$

where

56 $$\begin{aligned} {\left\{ \begin{array}{ll} &{}1<p\le \displaystyle \frac{4}{3},\\ &{}-1+p\le q<-2+2p, \end{array}\right. }\qquad \vee \qquad {\left\{ \begin{array}{ll} &{}\displaystyle \frac{4}{3}<p\le \displaystyle \frac{3}{2},\\ &{}-1+p\le q\le 2-p. \end{array}\right. } \end{aligned}$$

For a proof that $\rho _{qp}$ is indeed PPT entangled, see Appendix D.

## Conclusions

In this paper, we used a wide class of symmetric measurements to construct a family of positive, trace-preserving maps as well as the corresponding entanglement witnesses. Next, it is shown that our construction belongs to the family of witnesses that are based on the CCNR separability criterion^46^. The entalglement witnesses from symmetric measurements are recovered for a block-diagonal orthogonal matrix and the Hermitian orthonormal basis consisting in traceless operators and the identity. Several examples are provided using different sets of (*N*, *M*)-POVMs constructed from different operator bases. There remains an open question of generalizing our results to bipartite systems of different dimensions or even to a multipartite scenario.

## Supplementary Information

Supplementary Information.

## Data Availability

All data generated or analysed during this study are included in this published article and its supplementary information files.
