# Supplementary material for: Indecomposability of entanglement witnesses constructed from symmetric measurements
Source: Sci Rep. 2022 Jun 24;12:10785. doi: 10.1038/s41598-022-14920-5 (PMC9232524; doi:10.1038/s41598-022-14920-5)
Supplement: Supplementary file 1 — Supplementary Information. [file 41598_2022_14920_MOESM1_ESM.pdf]

Indecomposability of entanglement witnesses constructed from symmetric measurements

Katarzyna Siudzińska

Institute of Physics, Faculty of Physics, Astronomy and Informatics
Nicolaus Copernicus University in Toruń, ul. Grudziądzka 5, 87–100 Toruń, Poland

May 19, 2022

Appendix A Gell-Mann matrices

In $d=3$, a popular choice of a Hermitian orthonormal basis is the Gell-Mann matrices:

$$g_{01}=\frac{1}{\sqrt{2}}\begin{pmatrix}0&1&0\\1&0&0\\0&0&0\end{pmatrix},\qquad g_{10}=\frac{1}{\sqrt{2}}\begin{pmatrix}0&-i&0\\i&0&0\\0&0&0\end{pmatrix},$$
$$g_{02}=\frac{1}{\sqrt{2}}\begin{pmatrix}0&0&1\\0&0&0\\1&0&0\end{pmatrix},\qquad g_{20}=\frac{1}{\sqrt{2}}\begin{pmatrix}0&0&-i\\0&0&0\\i&0&0\end{pmatrix},$$
$$g_{12}=\frac{1}{\sqrt{2}}\begin{pmatrix}0&0&0\\0&0&1\\0&1&0\end{pmatrix},\qquad g_{21}=\frac{1}{\sqrt{2}}\begin{pmatrix}0&0&0\\0&0&-i\\0&i&0\end{pmatrix},$$
$$g_{11}=\frac{1}{\sqrt{2}}\begin{pmatrix}1&0&0\\0&-1&0\\0&0&0\end{pmatrix},\qquad g_{22}=\frac{1}{\sqrt{6}}\begin{pmatrix}1&0&0\\0&1&0\\0&0&-2\end{pmatrix},$$

and $G_0=\mathbb{I}/\sqrt{3}$. For the entanglement witness in Example 3, we fix the indices of $G_{\alpha,k}$ as follows,

$$\begin{aligned}G_{1,1}&=g_{01}, & G_{1,2}&=g_{10}, & G_{2,1}&=g_{02}, & G_{2,2}&=g_{20},\\ G_{3,1}&=g_{12}, & G_{3,2}&=g_{21}, & G_{4,1}&=g_{11}, & G_{4,2}&=g_{22}.\end{aligned} \tag{1}$$

In Example 5, on the other hand, we take

$$\begin{aligned}G_{1,1}&=g_{01}, & G_{1,2}&=g_{02}, & G_{1,3}&=g_{10}, & G_{1,4}&=g_{20},\\ G_{2,1}&=g_{12}, & G_{2,2}&=g_{21}, & G_{2,3}&=g_{11}, & G_{2,4}&=g_{22}.\end{aligned} \tag{2}$$

Appendix B   Hermitian orthonormal basis from MUBs

Using the complete set of four mutually unbiased bases in $d = 3$ and the corresponding projectors

$$
\begin{aligned}
E_{1,1} &= \begin{pmatrix} 1 & 0 & 0 \\ 0 & 0 & 0 \\ 0 & 0 & 0 \end{pmatrix}, &
E_{2,1} &= \frac{1}{3}\begin{pmatrix} 1 & 1 & 1 \\ 1 & 1 & 1 \\ 1 & 1 & 1 \end{pmatrix}, &
E_{3,1} &= \frac{1}{3}\begin{pmatrix} 1 & \omega^2 & \omega^2 \\ \omega & 1 & 1 \\ \omega & 1 & 1 \end{pmatrix}, &
E_{4,1} &= \frac{1}{3}\begin{pmatrix} 1 & \omega & \omega \\ \omega^2 & 1 & 1 \\ \omega^2 & 1 & 1 \end{pmatrix}, \\
E_{1,2} &= \begin{pmatrix} 0 & 0 & 0 \\ 0 & 1 & 0 \\ 0 & 0 & 0 \end{pmatrix}, &
E_{2,2} &= \frac{1}{3}\begin{pmatrix} 1 & \omega^2 & \omega \\ \omega & 1 & \omega^2 \\ \omega^2 & \omega & 1 \end{pmatrix}, &
E_{3,2} &= \frac{1}{3}\begin{pmatrix} 1 & \omega & 1 \\ \omega^2 & 1 & \omega^2 \\ 1 & \omega & 1 \end{pmatrix}, &
E_{4,2} &= \frac{1}{3}\begin{pmatrix} 1 & \omega^2 & 1 \\ \omega & 1 & \omega \\ 1 & \omega^2 & 1 \end{pmatrix}, \\
E_{1,3} &= \begin{pmatrix} 0 & 0 & 0 \\ 0 & 0 & 0 \\ 0 & 0 & 1 \end{pmatrix}, &
E_{2,3} &= \frac{1}{3}\begin{pmatrix} 1 & \omega & \omega^2 \\ \omega^2 & 1 & \omega \\ \omega & \omega^2 & 1 \end{pmatrix}, &
E_{3,3} &= \frac{1}{3}\begin{pmatrix} 1 & 1 & \omega \\ 1 & 1 & \omega \\ \omega^2 & \omega^2 & 1 \end{pmatrix}, &
E_{4,3} &= \frac{1}{3}\begin{pmatrix} 1 & 1 & \omega^2 \\ 1 & 1 & \omega^2 \\ \omega & \omega & 1 \end{pmatrix},
\end{aligned}
\tag{3}
$$

where $\omega = \exp(2\pi i/3)$, one finds the corresponding Hermitian orthonormal basis:

$$
\begin{aligned}
G_{1,1} &= \frac{1}{\sqrt{3}(1+\sqrt{3})}\begin{pmatrix} -2-\sqrt{3} & 0 & 0 \\ 0 & 1 & 0 \\ 0 & 0 & 1+\sqrt{3} \end{pmatrix}, &
G_{1,2} &= \frac{1}{\sqrt{3}(1+\sqrt{3})}\begin{pmatrix} 1 & 0 & 0 \\ 0 & -2-\sqrt{3} & 0 \\ 0 & 0 & 1+\sqrt{3} \end{pmatrix}, \\
G_{2,1} &= \frac{1}{2\sqrt{3}(1+\sqrt{3})}\begin{pmatrix} 0 & -v^* & -v \\ -v & 0 & -v^* \\ -v^* & -v & 0 \end{pmatrix}, &
G_{2,2} &= \frac{1}{\sqrt{3}(1+\sqrt{3})}\begin{pmatrix} 0 & iv^* & -iv \\ -iv & 0 & iv^* \\ iv^* & -iv & 0 \end{pmatrix}, \\
G_{3,1} &= \frac{1}{2\sqrt{3}(1+\sqrt{3})}\begin{pmatrix} 0 & u^* & iv^* \\ u & 0 & -v^* \\ -iv & -v & 0 \end{pmatrix}, &
G_{3,2} &= \frac{1}{\sqrt{3}(1+\sqrt{3})}\begin{pmatrix} 0 & u & -v^* \\ u^* & 0 & iv^* \\ -v & -iv & 0 \end{pmatrix}, \\
G_{4,1} &= \frac{1}{2\sqrt{3}(1+\sqrt{3})}\begin{pmatrix} 0 & u & -iv \\ u^* & 0 & -v \\ iv^* & -v^* & 0 \end{pmatrix}, &
G_{4,2} &= \frac{1}{\sqrt{3}(1+\sqrt{3})}\begin{pmatrix} 0 & u^* & -v \\ u & 0 & -iv \\ -v^* & iv^* & 0 \end{pmatrix},
\end{aligned}
$$

and $G_0 = \mathbb{I}/\sqrt{3}$, where $u = (1-i)(1+\sqrt{3})$ and $v = 2+\sqrt{3}+i$. The entanglement witness in Example 4 is given by eq. (40) with G_μ grouped in the following way,

$$
\{G_1, G_2, G_3\} = \{G_{1,2}, G_{2,1}, G_{2,2}\}, \qquad \{G_4, G_5, G_6, G_7, G_8\} = \{G_{1,1}, G_{3,1}, G_{3,2}, G_{4,1}, G_{4,2}\}. \tag{4}
$$

Appendix C   States with positive partial transposition in $d = 3$

In Examples 3-5, we propose PPT states ρ_1, ρ_2, and ρ_3 that are detected by the corresponding entanglement witnesses $\widetilde{W}_1$, $\widetilde{W}_2$, and $\widetilde{W}_3'$. Here, we show that these states are indeed PPT entangled. First, observe that the eigenvalues of these states are positive,

$$
\begin{aligned}
\lambda(\rho_1) &= \Big\{\eta_1, \eta_2, \frac{125}{579}, \frac{125}{579}, \frac{125}{579}, \frac{34}{579}, \frac{34}{579}, \frac{34}{579}, \eta_3\Big\}, \\
\lambda(\rho_2) &= \Big\{\frac{2}{7}, \frac{2}{7}, \frac{5}{21}, \frac{2}{21}, \frac{2}{21}, 0, 0, 0, 0\Big\}, \\
\lambda(\rho_3) &= \Big\{\frac{10+3\sqrt{5}}{90}, \frac{10+3\sqrt{5}}{90}, \frac{1}{9}, \frac{1}{9}, \frac{1}{9}, \frac{1}{9}, \frac{10-3\sqrt{5}}{90}, \frac{10-3\sqrt{5}}{90}\Big\},
\end{aligned}
\tag{5}
$$

where $\eta_1 \simeq 0.394$, $\eta_2 \simeq 0.245$, $\eta_3 \simeq 0.00840$. Moreover, $\mathrm{Tr}\rho_k = 1$, $k = 1,2,3$, and hence ρ_k, are legitimate quantum states. Partial transpositions of ρ_k are represented by the following matrices,

$$\rho_1^\Gamma = \frac{1}{579}\left[\begin{array}{ccc|ccc|ccc} 125 & \cdot & \cdot & \cdot & \cdot & \cdot & \cdot & \cdot & \cdot \\ \cdot & 125 & \cdot & \nu & \cdot & \cdot & \cdot & \cdot & \cdot \\ \cdot & \cdot & 34 & \cdot & \cdot & \cdot & \nu & \cdot & \cdot \\ \hline \cdot & \overline{\nu} & \cdot & 34 & \cdot & \cdot & \cdot & \cdot & \cdot \\ \cdot & \cdot & \cdot & \cdot & 125 & \cdot & \cdot & \cdot & \cdot \\ \cdot & \cdot & \cdot & \cdot & \cdot & 125 & \cdot & \nu & \cdot \\ \hline \cdot & \cdot & \overline{\nu} & \cdot & \cdot & \cdot & 125 & \cdot & \cdot \\ \cdot & \cdot & \cdot & \cdot & \cdot & \overline{\nu} & \cdot & 34 & \cdot \\ \cdot & \cdot & \cdot & \cdot & \cdot & \cdot & \cdot & \cdot & 125 \end{array}\right], \qquad \nu = -5(5-12i), \tag{6}$$

$$\rho_2 = \frac{1}{21}\left[\begin{array}{ccc|ccc|ccc} 3 & \cdot & \cdot & \cdot & \cdot & 2 & \cdot & 2 & \cdot \\ \cdot & 2 & \cdot & 1 & \cdot & \cdot & \cdot & \cdot & 2 \\ \cdot & \cdot & 2 & \cdot & 2 & \cdot & 1 & \cdot & \cdot \\ \hline \cdot & 1 & \cdot & 2 & \cdot & \cdot & \cdot & \cdot & 2 \\ \cdot & \cdot & 2 & \cdot & 3 & \cdot & 2 & \cdot & \cdot \\ 2 & \cdot & \cdot & \cdot & \cdot & 2 & \cdot & 1 & \cdot \\ \hline \cdot & \cdot & 1 & \cdot & 2 & \cdot & 2 & \cdot & \cdot \\ 2 & \cdot & \cdot & \cdot & \cdot & 1 & \cdot & 2 & \cdot \\ \cdot & 2 & \cdot & 2 & \cdot & \cdot & \cdot & \cdot & 3 \end{array}\right], \tag{7}$$

$$\rho_3^\Gamma = \frac{1}{90}\left[\begin{array}{ccc|ccc|ccc} 10 & \cdot & \cdot & \cdot & 3-6i & \cdot & \cdot & \cdot & -3-6i \\ \cdot & 10 & \cdot & \cdot & \cdot & \cdot & \cdot & \cdot & \cdot \\ \cdot & \cdot & 10 & \cdot & \cdot & \cdot & \cdot & \cdot & \cdot \\ \hline \cdot & \cdot & \cdot & 10 & \cdot & \cdot & \cdot & \cdot & \cdot \\ 3+6i & \cdot & \cdot & \cdot & 10 & \cdot & \cdot & \cdot & \cdot \\ \cdot & \cdot & \cdot & \cdot & \cdot & 10 & \cdot & \cdot & \cdot \\ \hline \cdot & \cdot & \cdot & \cdot & \cdot & \cdot & 10 & \cdot & \cdot \\ \cdot & \cdot & \cdot & \cdot & \cdot & \cdot & \cdot & 10 & \cdot \\ -3+6i & \cdot & \cdot & \cdot & \cdot & \cdot & \cdot & \cdot & 10 \end{array}\right], \tag{8}$$

and their eigenvalues are again positive, which can be seen from

$$\begin{aligned} \lambda(\rho_1^\Gamma) &= \Big\{\frac{159+13\sqrt{149}}{1158}, \frac{159+13\sqrt{149}}{1158}, \frac{159+13\sqrt{149}}{1158}, \frac{125}{579}, \frac{125}{579}, \frac{125}{579}, \frac{159-13\sqrt{149}}{1158}, \\ &\qquad \frac{159-13\sqrt{149}}{1158}, \frac{159-13\sqrt{149}}{1158}\Big\}, \\ \lambda(\rho_2^\Gamma) &= \Big\{\frac{3+2\sqrt{2}}{21}, \frac{3+2\sqrt{2}}{21}, \frac{3+2\sqrt{2}}{21}, \frac{1}{21}, \frac{1}{21}, \frac{1}{21}, \frac{3-2\sqrt{2}}{21}, \frac{3-2\sqrt{2}}{21}, \frac{3-2\sqrt{2}}{21}\Big\}, \\ \lambda(\rho_3^\Gamma) &= \Big\{\frac{10+3\sqrt{10}}{90}, \frac{1}{9}, \frac{1}{9}, \frac{1}{9}, \frac{1}{9}, \frac{1}{9}, \frac{1}{9}, \frac{1}{9}, \frac{10-3\sqrt{10}}{90}\Big\}. \end{aligned} \tag{9}$$

Thus, ρ_k have positive partial transpositions. Finally, observe that these states are indeed detected via the corresponding witnesses due to

$$\mathrm{Tr}(\widetilde{W}_1\rho_1) = \frac{311-180\sqrt{3}}{579} \simeq -0.00133, \qquad \mathrm{Tr}(\widetilde{W}_2\rho_2) = -\frac{1}{21}, \qquad \mathrm{Tr}(\widetilde{W}_3'\rho_3) = -1 - \frac{\sqrt{5}}{3}. \tag{10}$$

Appendix D Family of PPT states in $d = 4$

In Section 4, we consider the two-parameter family of quantum states

$$\rho_{qp} = \frac{1}{8(q+4)} \left[\begin{array}{cccc|cccc|cccc|cccc}
q+2 & \cdot & \cdot & \cdot & \cdot & q & \cdot & \cdot & \cdot & \cdot & p & \cdot & \cdot & \cdot & \cdot & p \\
\cdot & 2 & \cdot & \cdot & \cdot & \cdot & \cdot & \cdot & \cdot & \cdot & \cdot & \cdot & \cdot & \cdot & \cdot & \cdot \\
\cdot & \cdot & q+2 & \cdot & \cdot & \cdot & \cdot & q & p & \cdot & \cdot & \cdot & \cdot & p & \cdot & \cdot \\
\cdot & \cdot & \cdot & 2 & \cdot & \cdot & \cdot & \cdot & \cdot & \cdot & \cdot & \cdot & \cdot & \cdot & \cdot & \cdot \\
\hline
\cdot & \cdot & \cdot & \cdot & 2 & \cdot & \cdot & \cdot & \cdot & \cdot & \cdot & \cdot & \cdot & \cdot & \cdot & \cdot \\
q & \cdot & \cdot & \cdot & \cdot & q+2 & \cdot & \cdot & \cdot & \cdot & p & \cdot & \cdot & \cdot & \cdot & p \\
\cdot & \cdot & \cdot & \cdot & \cdot & \cdot & 2 & \cdot & \cdot & \cdot & \cdot & \cdot & \cdot & \cdot & \cdot & \cdot \\
\cdot & \cdot & q & \cdot & \cdot & \cdot & \cdot & q+2 & p & \cdot & \cdot & \cdot & \cdot & p & \cdot & \cdot \\
\hline
\cdot & \cdot & p & \cdot & \cdot & \cdot & \cdot & p & q+2 & \cdot & \cdot & \cdot & \cdot & q & \cdot & \cdot \\
\cdot & \cdot & \cdot & \cdot & \cdot & \cdot & \cdot & \cdot & \cdot & 2 & \cdot & \cdot & \cdot & \cdot & \cdot & \cdot \\
p & \cdot & \cdot & \cdot & \cdot & p & \cdot & \cdot & \cdot & \cdot & q+2 & \cdot & \cdot & \cdot & \cdot & q \\
\cdot & \cdot & \cdot & \cdot & \cdot & \cdot & \cdot & \cdot & \cdot & \cdot & \cdot & 2 & \cdot & \cdot & \cdot & \cdot \\
\hline
\cdot & \cdot & \cdot & \cdot & \cdot & \cdot & \cdot & \cdot & \cdot & \cdot & \cdot & \cdot & 2 & \cdot & \cdot & \cdot \\
\cdot & \cdot & p & \cdot & \cdot & \cdot & \cdot & p & q & \cdot & \cdot & \cdot & \cdot & q+2 & \cdot & \cdot \\
\cdot & \cdot & \cdot & \cdot & \cdot & \cdot & \cdot & \cdot & \cdot & \cdot & \cdot & \cdot & \cdot & \cdot & 2 & \cdot \\
p & \cdot & \cdot & \cdot & \cdot & p & \cdot & \cdot & \cdot & \cdot & q & \cdot & \cdot & \cdot & \cdot & q+2
\end{array}\right]. \tag{11}$$

Onserve that ρ_{qp} is trace preserving by construction, and its eigenvalues

$$\lambda(\rho_{qp}) \in \left\{ \frac{1}{4(q+4)}, \frac{1+q \pm p}{4(q+4)} \right\} \tag{12}$$

are positive if and only if

$$\left\{ \begin{array}{l} p \geq 0, \\ q \geq -1+p, \end{array} \right. \quad \vee \quad \left\{ \begin{array}{l} p < 0, \\ q \geq -1-p. \end{array} \right. \tag{13}$$

Now, the partial transposition of ρ_{qp} is given by

$$\rho_{qp}^{\Gamma} = \frac{1}{8(q+4)} \left[\begin{array}{cccc|cccc|cccc|cccc}
q+2 & \cdot & \cdot & \cdot & \cdot & \cdot & \cdot & \cdot & \cdot & \cdot & p & \cdot & \cdot & \cdot & \cdot & \cdot \\
\cdot & 2 & \cdot & \cdot & q & \cdot & \cdot & \cdot & \cdot & \cdot & \cdot & \cdot & \cdot & \cdot & p & \cdot \\
\cdot & \cdot & q+2 & \cdot & \cdot & \cdot & \cdot & \cdot & p & \cdot & \cdot & \cdot & \cdot & \cdot & \cdot & \cdot \\
\cdot & \cdot & \cdot & 2 & \cdot & \cdot & q & \cdot & \cdot & \cdot & \cdot & \cdot & p & \cdot & \cdot & \cdot \\
\hline
\cdot & q & \cdot & \cdot & 2 & \cdot & \cdot & \cdot & \cdot & \cdot & \cdot & p & \cdot & \cdot & \cdot & \cdot \\
\cdot & \cdot & \cdot & \cdot & \cdot & q+2 & \cdot & \cdot & \cdot & \cdot & \cdot & \cdot & \cdot & \cdot & \cdot & p \\
\cdot & \cdot & \cdot & q & \cdot & \cdot & 2 & \cdot & \cdot & p & \cdot & \cdot & \cdot & \cdot & \cdot & \cdot \\
\cdot & \cdot & \cdot & \cdot & \cdot & \cdot & \cdot & q+2 & \cdot & \cdot & \cdot & \cdot & \cdot & p & \cdot & \cdot \\
\hline
\cdot & \cdot & p & \cdot & \cdot & \cdot & \cdot & \cdot & q+2 & \cdot & \cdot & \cdot & \cdot & \cdot & \cdot & \cdot \\
\cdot & \cdot & \cdot & \cdot & \cdot & \cdot & p & \cdot & \cdot & 2 & \cdot & \cdot & q & \cdot & \cdot & \cdot \\
p & \cdot & \cdot & \cdot & \cdot & \cdot & \cdot & \cdot & \cdot & \cdot & q+2 & \cdot & \cdot & \cdot & \cdot & \cdot \\
\cdot & \cdot & \cdot & \cdot & p & \cdot & \cdot & \cdot & \cdot & \cdot & \cdot & 2 & \cdot & \cdot & q & \cdot \\
\hline
\cdot & \cdot & \cdot & p & \cdot & \cdot & \cdot & \cdot & \cdot & q & \cdot & \cdot & 2 & \cdot & \cdot & \cdot \\
\cdot & \cdot & \cdot & \cdot & \cdot & \cdot & \cdot & p & \cdot & \cdot & \cdot & \cdot & \cdot & q+2 & \cdot & \cdot \\
\cdot & p & \cdot & \cdot & \cdot & \cdot & \cdot & \cdot & \cdot & \cdot & \cdot & q & \cdot & \cdot & 2 & \cdot \\
\cdot & \cdot & \cdot & \cdot & \cdot & p & \cdot & \cdot & \cdot & \cdot & \cdot & \cdot & \cdot & \cdot & \cdot & q+2
\end{array}\right], \tag{14}$$

and the corresponding eigenvalues

$$\lambda(\rho_{qp}^{\Gamma}) \in \left\{ \frac{2 \pm (q+p)}{8(q+4)}, \frac{2 \pm (q-p)}{8(q+4)} \right\} \tag{15}$$

are positive (that is, ρ_{qp} PPT) if and only if

$$\begin{cases} -2 \leq p \leq 0, \\ -2 - p \leq q < 2 + p \end{cases} \vee \begin{cases} 0 < p \leq 2, \\ -2 + p \leq q \leq 2 - p. \end{cases} \tag{16}$$

Finally, quantum entanglement of the PPT state ρ_{qp} is detected via $\widetilde{W}_4$ only when

$$\mathrm{Tr}(\rho_{qp}\widetilde{W}_4) = \frac{3}{2}\frac{2(1-p)+q}{q+4} < 0, \tag{17}$$

which, together with the earlier conditions for the parameters p and q, results in eq. (56) in the main text.
